# Metabolic reprogramming of fetal hematopoietic stem and progenitor cells by maternal obesity

**DOI:** 10.3389/frhem.2025.1575143

**Published:** 2025-04-14

**Authors:** Oleg Varlamov

**Affiliations:** Division of Metabolic Health and Disease, Oregon National Primate Research Center, Beaverton, OR, United States

**Keywords:** bone marrow adipocytes, fetal development, hematopoiesis, hematopoietic stem and progenitor cells, maternal obesity, western-style diet, nonhuman primates, programming

## Abstract

Maternal obesity, often linked to the consumption of a high-fat Western-style diet (WSD), poses significant risks to both maternal and fetal health. This review explores the impact of maternal obesity on fetal hematopoietic stem and progenitor cells (HSPCs), highlighting how metabolic and inflammatory shifts in the maternal environment affect HSPC proliferation, differentiation, and long-term immune system development. Maternal obesity leads to hormonal imbalances, increased inflammatory cytokines, placental insufficiency, and altered nutrient availability that disrupt normal HSPC function, potentially predisposing offspring to immune dysfunction, metabolic disorders, and cardiovascular diseases later in life. Notably, maternal obesity skews HSPC differentiation toward the myeloid lineage, which can impair adaptive immune responses and increase the risk of autoimmune diseases and infections. Furthermore, maternal diet-driven epigenetic and transcriptional reprogramming of fetal HSPCs exacerbates chronic inflammation, reinforcing a pro-inflammatory phenotype in downstream progeny that persists into postnatal stages. The review also emphasizes the need for further research to clarify the mechanisms underlying these effects across different species and developmental stages, as well as the potential for targeted interventions to mitigate the adverse impacts of maternal obesity on fetal hematopoiesis and lifelong health outcomes.

## Introduction

Maternal obesity has emerged as a significant public health concern, driven by a complex interplay of dietary habits, lifestyle choices, and genetic predispositions. The prevalence of obesity, particularly linked to the consumption of high-fat Western-style diet (WSD) characterized by high caloric intake and low nutritional quality, has increased dramatically in recent decades. In the United States, more than 40% of pregnant women are classified as obese in 2019 (1), with prepregnancy obesity rates rising with ~11% increase in prevalence between 2016 and 2019 (2). This condition poses substantial risks not only to maternal health but also to the developing fetus, with obesity prior to pregnancy identified as a critical risk factor for a range of maternal and fetal complications. Specifically, maternal obesity significantly increases the risk of gestational diabetes (GD) mellitus, which can lead to excessive fetal growth (macrosomia) and complications during delivery (3-5). Maternal obesity is also linked to a higher incidence of hypertensive disorders, such as gestational hypertension and preeclampsia, which can result in serious complications including placental abruption and preterm birth (6, 7). Maternal obesity is also associated with an increased risk of stillbirth and can contribute to long-term health implications for offspring, including obesity, metabolic syndrome, and cardiovascular diseases (8-18).

Moreover, children born to obese mothers often exhibit immunological complications, such as reduced immune responses to ex vivo stimulation with toll-like receptor (TLR) ligands (12, 19) and bacterial/viral pathogens, which increase their susceptibility to infections (20). Studies have shown that these children may have altered immune cell development, leading to a higher risk of conditions like asthma and other allergic diseases (21, 22). The dysregulation of innate immunity in early life can have long-term consequences on metabolic diseases and behavioral disorders, further complicating their health outcomes. Despite the growing body of literature addressing these implications, there remains a notable gap in our understanding of how maternal obesity affects developmental hematopoiesis—the process by which blood cells are formed during fetal development. Limited data exist on how maternal obesity influences this critical aspect of fetal growth, highlighting the need for further research to elucidate the mechanisms involved and the potential long-term consequences for offspring.

This review will specifically focus on primitive hematopoietic stem cells (HSCs) and hematopoietic progenitor cells (HPCs), collectively referred to as hematopoietic stem and progenitor cells (HSPCs). It will examine both sex-specific and species-specific aspects of developmental hematopoiesis, including the impact of maternal obesity and gestational diabetes mellitus on HSPC proliferation, differentiation, metabolic programming, mitochondrial function, and overall functional characteristics. Additionally, the impact of maternal obesity on the immune system has been addressed in other review articles (22-25). The effects of obesity on hematopoiesis and the bone marrow niche in adults have been detailed in several excellent reviews (26-30).

## Developmental hematopoiesis and bone marrow niche formation

**FIGURE 1:**
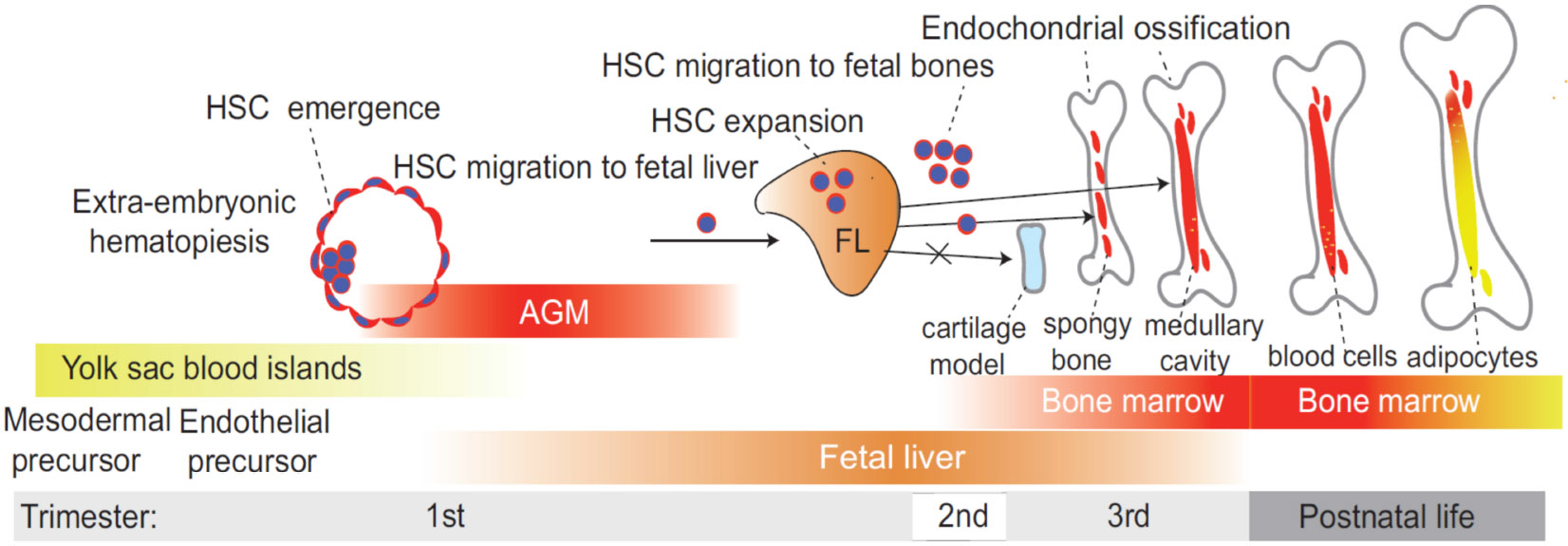
Timeline of developmental hematopoiesis. During fetal development, hematopoiesis occurs in several distinct waves, involving the sequential migration of hematopoietic stem cells (HSC) between different tissues. Initially, HSC emerge in the aorta-gonad-mesonephros (AGM) region during the first trimester, marking the first site of definitive hematopoiesis. As development progresses, the fetal liver (FL) becomes the primary hematopoietic niche during the second trimester, serving as the main site for HSC expansion. In the early third trimester, HSCs begin transitioning from the FL to the fetal bone marrow, where they establish the long-term hematopoietic system that persists into adulthood. This relocation coincides with vascular development, bone ossification, and the formation of the central marrow cavity, all of which are essential for creating a functional bone marrow microenvironment that supports lifelong hematopoiesis. Bone marrow adipocytes emerge during postnatal development.

Developmental hematopoiesis is a complex and dynamic process that involves the formation of HPCs and mature blood cells from HSCs (Figure 1). This process is crucial for establishing a functional hematopoietic system, which is essential for maintaining homeostasis and responding to physiological demands throughout an organism’s life. During fetal development, hematopoiesis occurs in several waves that involve the migration of HSCs between different tissues. Several review articles provide a detailed discussion on developmental hematopoiesis (31-35). Initially, hematopoiesis takes place in the yolk sac, where primitive hematopoiesis generates early red blood cells and immune cells (36-38). During this stage, the primary immune cells produced are primitive macrophages, which play a role in early immune responses (39, 40). These cells are short-lived and are eventually replaced by definitive erythrocytes produced later in development (41). Following the emergence of definitive HSPCs in the aorta gonad mesonephros (AGM) during the first trimester, the fetal liver becomes the main embryonic niche for HSC expansion during the second trimester (42, 43). During the early third trimester, HSCs begin relocating from the fetal liver (FL) to the fetal bone marrow (34). The regulation of developmental hematopoiesis involves a variety of intrinsic and extrinsic factors. The bone marrow microenvironment, including adipocytes, osteolineage, stromal cells, megakaryocytes and endothelial cells, provides essential paracrine signals that support HSC maintenance and function (33, 44-49). This emergence of HSCs in the fetal bone marrow coincides with the development of the fetal bone marrow vascular system, bone ossification, and formation of the central marrow cavity (50, 51) (Figure 1).

Bone marrow adipogenesis begins postnatally, with the first adipocytes appearing in the distal skeleton (e.g., long bones like the tibia and femur) and gradually expanding to more central regions (e.g., vertebrae, sternum and ribs) with age (28, 52) (Figure 1). While bone marrow adipogenesis is typically postnatal, the premature appearance of bone marrow adipocytes during prenatal development may have pathological impacts on immune cell development in the fetus. The role of bone marrow adipocytes in hematopoiesis regulation remains contentious. Evidence from two studies suggests that bone marrow adipocytes act as negative regulators of hematopoiesis *in vivo*. In an initial study, Naveiras et al. demonstrated that fatless mice or wild-type mice treated with a peroxisome proliferator-activated receptor-gamma (PPARγ) inhibitor, which inhibits adipogenesis, exhibited enhanced HSC engraftment following irradiation (53). Additionally, the absence of bone marrow adipocytes in fatless mice led to a compensatory increase in osteogenesis after hematopoietic ablation. These findings were later corroborated by another study, which showed that bone marrow adipocytes suppress both hematopoietic recovery and osteogenesis post-irradiation (54). Since transplanted HSCs predominantly home near endosteal bone surfaces following bone marrow ablation (35), it is possible that bone marrow adipocytes indirectly inhibit HSC engraftment by suppressing osteoblast differentiation. Given that osteoblasts and adipocytes originate from a common bone marrow mesenchymal progenitor population (52), increased adipogenesis may directly impair osteogenesis, thereby reducing HSC engraftment. In addition to their effects on osteogenesis, bone marrow adipocytes may also influence HSC function through local paracrine signaling. For instance, studies in mice have demonstrated that bone marrow adipocytes produce stem cell factor (SCF), which plays a crucial role in hematopoietic regeneration following irradiation or chemotherapy (55) and in restoring myelopoiesis after metabolic stress induced by a high-fat diet (HFD) (56).

In line with these findings, human acute myeloid leukemia has been shown to disrupt the bone marrow adipocyte niche, leading to impaired myelo-erythropoiesis, while *in vivo* administration of PPARγ agonists stimulated bone marrow adipogenesis and reversed leukemia-induced hematopoietic failure (57). Notably, both obesity (30) and aging (58) are associated with increased bone marrow adipogenesis, coinciding with a shift toward myeloid-biased hematopoiesis. As of today, there are no comprehensive reports detailing developmental bone marrow adipogenesis. Studies in non-human primates (NHPs) showed that maternal obesity can induce an adipogenic environment in the fetal bone marrow, characterized by an increase in adipocyte numbers and size (59, 60). This change can disrupt the bone marrow niche, which is essential for maintaining HSC function. Therefore, our understanding is limited to the postnatal context. Future studies on prenatal adipogenesis may uncover new insights into the links between early adipocyte development and immune system formation.

## Impact of maternal obesity on HSPC number, proliferation and differentiation

**FIGURE 2:**
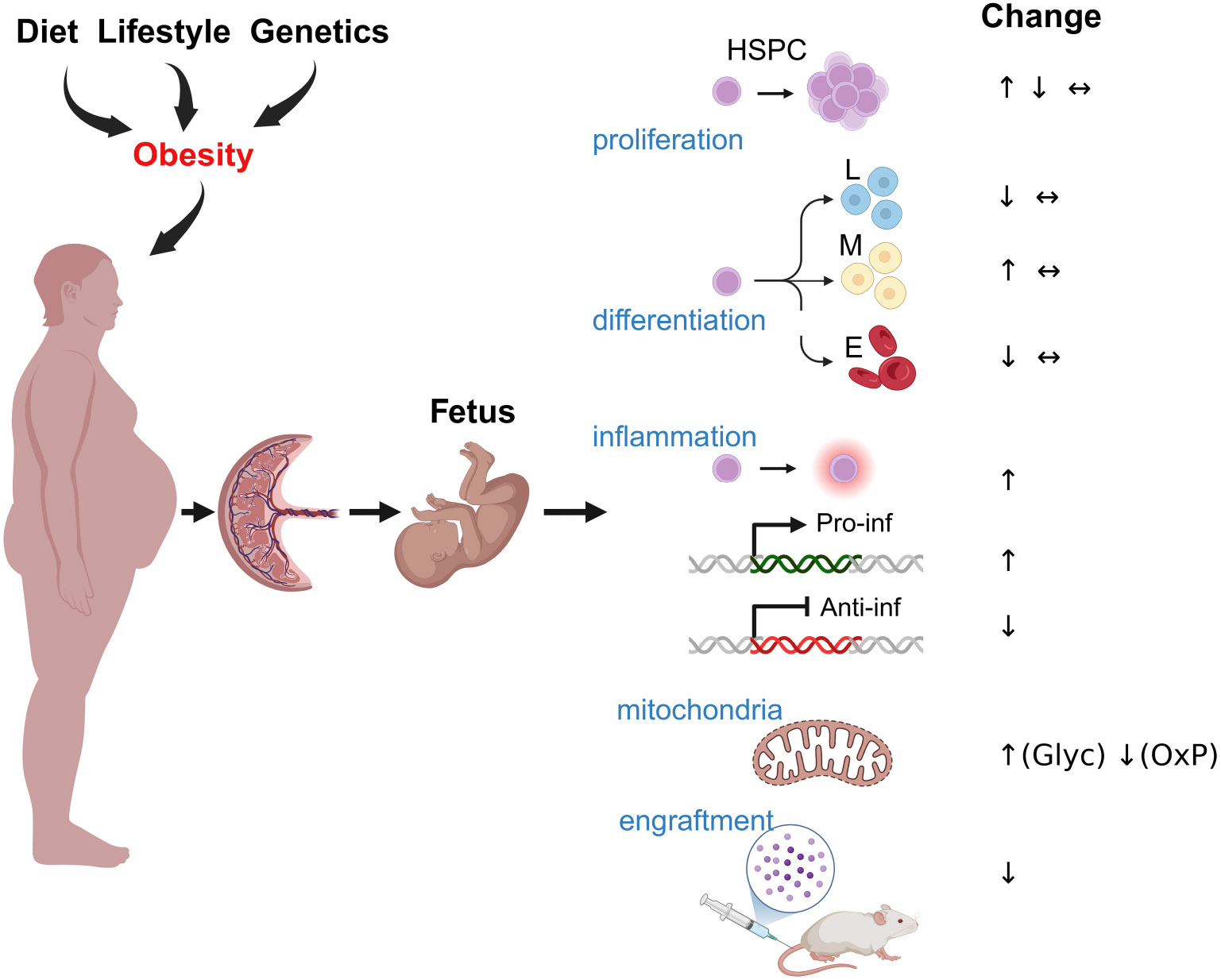
The impact of maternal obesity on HSPCs. Maternal obesogenic diet, lifestyle, and genetic factors can influence fetal development through several potential mechanisms, including inflammation, nutrient alterations, hormonal imbalance, maternal microbiome dysregulation, and placental insufficiency. These factors may directly affect HSPC function and regulation, leading to changes in proliferation rates and HSPC numbers, the development of myeloid-biased HSPC differentiation at the expense of lymphoid and erythroid differentiation, activation of a proinflammatory program in HSPCs, and reduced expression of anti-inflammatory factors. Additionally, they can promote increased glycolysis at the expense of oxidative phosphorylation and impair the regenerative properties of HSPCs *in vivo*.

The impact of maternal obesity on fetal HSCs and their progeny is not fully understood, but several studies have begun to elucidate its effects (Figure 2). One earlier study by Kamimae-Lanning et al. (61) demonstrated a reduction in the frequencies of primitive c-Kit+ Sca1+ HSCs in the fetal liver of mice exposed to diet-induced maternal obesity. Recent studies also demonstrated that male but not female offspring of obese dams showed a decrease in primitive bone marrow c-Kit+ Sca1+ HSC numbers and the development of glucose intolerance in mice (62). These findings align with studies in adult mice, which shows that diet-induced obesity leads to a shift in HSCs capable of self-renewal toward maturing multipotent progenitor cells. The increased differentiation potential in these cells results in enhanced myeloid ex vivo colony-forming capacity (63). This suggests that obesity stimulates the differentiation of HSCs while simultaneously reducing their proliferation, ultimately leading to a decreased HSC population (Figure 2).

In contrast, studies in NHPs demonstrated different and conflicting outcomes. Sureshchandra et al. found that maternal obesity was not associated with changes in the frequencies and in vitro colony-forming capacity of CD34+ HSPCs derived from the fetal bone marrow (60). In parallel, maternal WSD disrupted B-cell development by downregulating essential transcription factors (BCL11A, BTG2, HHEX) and cell adhesion molecules (CD164, ITGA4, ESAM) in common lymphoid progenitors (CLPs), leading to a significant reduction in fetal bone marrow B-cell numbers (60). The functional consequences of these changes were evident in transplantation studies using immunodeficient NOD/SCID/IL2rγ−/− mice. Thirteen weeks post-engraftment, fetal bone marrow CD34+ HSPCs from WSD-exposed fetuses showed significantly lower engraftment efficiency in mouse bone marrow compared to controls. While B-and T-cell frequencies remained unchanged, myeloid cell populations, including monocytes and granulocytes, were markedly reduced in the bone marrow of engrafted mice. Interestingly, colony-forming assays indicated no intrinsic differences in granulocyte-macrophage (CFU-GM) or erythroid (BFU-E) progenitor numbers, suggesting that maternal WSD primarily impaired *in vivo* differentiation and regenerative potential rather than altering intrinsic lineage commitment (60). However, another NHP study by Nash et al. reported that exposure to a maternal WSD resulted in increased frequencies of bone marrow HSPCs in both fetuses and juvenile offspring from obese dams (59). Interestingly, ex vivo erythroid colony-forming capacity was reduced in CD34+ HSPCs derived from obese dams (59). These contrasting findings highlight the complexity of maternal obesity’s effects on fetal HSPC proliferation and underscore the need for further research to clarify these relationships across different species, developmental stages, and hematopoietic tissues.

## Impact of maternal obesity on proinflammatory cytokines

**FIGURE 3:**
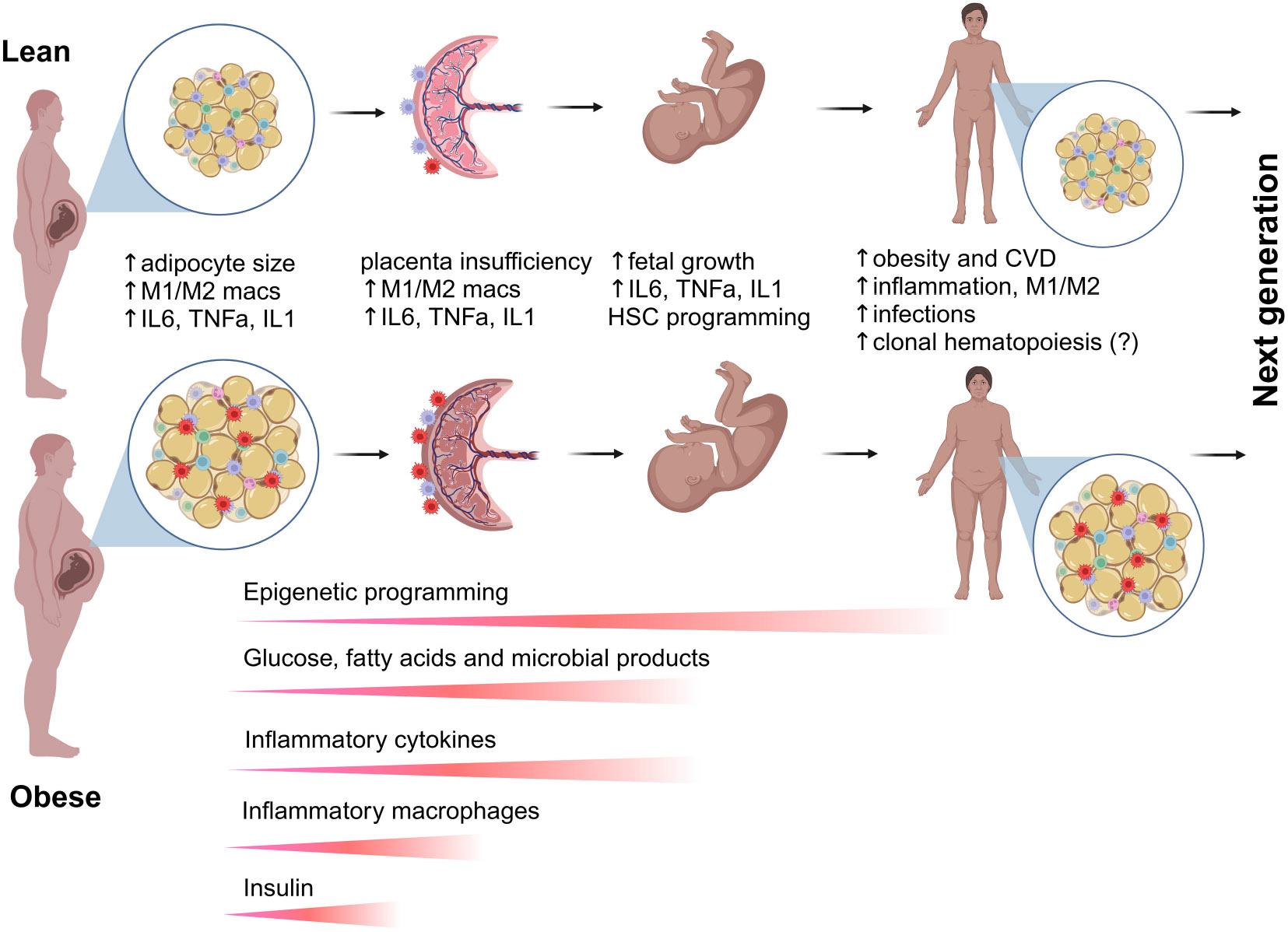
Transgenerational effects of maternal obesity on hematopoiesis and immunity. Maternal obesity is associated with excess body fat, insulin resistance, and dyslipidemia, leading to altered levels of circulating molecules, including glucose, fatty acids, and microbial metabolites. Additionally, maternal obesity induces a proinflammatory milieu in adipose tissue and the placenta, promoting the accumulation of proinflammatory M1 macrophages at the expense of alternatively activated M2 macrophages. Proinflammatory cytokines secreted by tissue-resident and circulating immune cells, along with metabolic factors, can cross the placenta and enter fetal circulation, directly influencing fetal growth and HSPC programming via epigenetic mechanisms. Disruptions in the placental immune environment can impair the maternal-fetal interface, leading to immune tolerance breakdown and inflammation. Both circulating and niche-specific factors affect HSPC development and fate commitment, resulting in dysregulated differentiation and abnormal seeding of peripheral tissues immune cells. Offspring of obese mothers are at an increased risk of obesity, which further influences HSPC development within their own lifetime. The combined effects of prenatal and postnatal obesogenic stimuli have lasting impacts on stem cells and their progeny, increasing the risk of obesity, chronic inflammation, infectious diseases, and potentially malignant hematopoiesis in future generations.

Maternal obesity alters the metabolic environment *in utero*, characterized by elevated levels of inflammatory cytokines (23, 64-66), altered nutrient availability, and alterations in the hormonal milieu, including changes in insulin, IGFI/II, leptin and adiponectin levels (67-69). These factors can significantly impact the fetal hematopoietic system (Figure 3). Studies have shown that a HFD can lead to a hyperinflammatory state in the fetal environment, which may disrupt normal HSPC differentiation (22, 25, 70). One of the primary proposed mechanisms through which maternal obesity affects HSPC differentiation is the elevation of inflammatory cytokines such as C-reactive protein (CRP), tumor necrosis factor-alpha (TNF-α), interleukin-6 (IL-6) and monocyte chemo-attractant protein-1 (MCP-1) (71-77), although the role of inflammatory cytokines in maternal obesity-induced inflammation remains controversial (65). Inflammatory cytokines can influence HSPC differentiation by promoting myeloid lineage commitment at the expense of lymphoid development and self-renewal (29, 78, 79). This shift can lead to an imbalance in immune cell populations, potentially increasing the risk of autoimmune diseases and infections in the offspring.

Theoretically, multiple factors can contribute to a hyperinflammatory fetal bone marrow microenvironment, similar to the inflammatory remodeling of the bone marrow observed in aging. For example, excess bone marrow adipose tissue can lead to altered proinflammatory signaling pathways in HSPCs (28, 30, 34). Moreover, maternal obesity can have a significant impact on the stromal cells that support HSPCs. Stromal cells are essential for maintaining the HSPC niche, where they provide signals that regulate the self-renewal, differentiation, and overall function of hematopoietic cells (34, 44, 47, 80). In aged bone marrow, inflammation is marked by the loss of osteoprogenitors at the endosteum, expansion of inflammatory mesenchymal stromal cells (MSCs), and deterioration of the sinusoidal vasculature. This chronic inflammatory state, primarily driven by interleukin-1β (IL-1β), leads to the persistent activation of emergency myelopoiesis, skewing HSC differentiation toward myeloid lineages and impairing regenerative and self-renewal capacity (78, 81). Given that blocking IL-1 signaling has been shown to mitigate these aging-related defects (78), targeting IL-1 may also represent a potential strategy to prevent maternal inflammation-induced alterations in fetal hematopoiesis.

Importantly, studies have suggested that proinflammatory cytokines play a crucial role in selecting for and promoting clonal hematopoiesis (CH). Increased levels of inflammatory cytokines and chemokines, such as IL-1β, IL-6, and TNF-α, have been strongly associated with CH in human studies (82-84). Chronic inflammation creates a selective pressure that favors the expansion of HSPCs harboring CH-associated mutations, as these mutant clones often exhibit resistance to inflammatory stress. This inflammatory-driven clonal expansion has been linked to an increased risk of hematologic malignancies and cardiovascular diseases, highlighting the significant interplay between systemic inflammation and CH progression (84). However, whether chronic maternal inflammation promotes CH and hematopoietic malignancies in the offspring remains unknown (Figure 3). The impacts of obesity on bone marrow inflammation and the mechanisms underlying enhanced myelopoiesis in adults have been extensively reviewed (29, 85, 86).

## Maternal obesity and the maternal-fetal interface

The dysregulation of the maternal-fetal interface can have lasting effects on fetal immune system development (Figure 3). However, the specific mechanisms remain largely unknown due to the intricate network of maternal signals originating from or transmitted through the placenta. Alterations in placental function, including disruptions in nutrient and oxygen transport, inflammatory signaling, and immune cell composition, can impact fetal hematopoiesis. During pregnancy, the maternal immune system faces the unique challenge of tolerating the genetically distinct fetus while still maintaining immune defenses. Instead of rejecting the fetus as a foreign entity, the maternal immune system undergoes significant adaptations, particularly at the maternal-fetal interface. This involves a delicate balance between immune tolerance and protective immunity (87). The detailed biology of the placental immune landscape is beyond the scope of this review and has been extensively discussed in several excellent review articles (87-91).

In brief, the maternal decidua, which lines the uterus during pregnancy, consists primarily of decidual natural killer cells, decidual macrophages, and T cells. These immune cells play a crucial role in maintaining maternal-fetal tolerance, facilitating placental development, and regulating immune responses at the maternal-fetal interface (87, 88). In contrast, the immune composition within the fetal chorionic villi is exclusively made up of myeloid cells, including fetal Hofbauer cells and placenta-associated maternal monocytes and macrophages (92). Hofbauer cells, which originate from the fetus, reside in the mesenchymal core of the chorionic villi and contribute to placental development, blood vessel formation, immune modulation, and defense against pathogens. Meanwhile, placenta-associated maternal monocytes represent a diverse population of maternal monocytes and macrophages that infiltrate the placenta, supporting immune regulation and tissue remodeling. Together, these maternal and fetal immune cells establish a carefully regulated environment that ensures proper fetal development while protecting against infections and excessive inflammation (87-91).

Extensive research has been conducted to elucidate the impact of maternal obesity on macrophages, revealing significant alterations in their function, polarization, and inflammatory responses. Macrophages are highly plastic immune cells that regulate maternal-fetal interactions, aiding in implantation, placental development, and immune tolerance [reviewed in (88-90)]. During early pregnancy, macrophages exhibit an M1-dominant phenotype to facilitate implantation. As pregnancy progresses, a mixed M1/M2 phenotype supports trophoblast invasion and vascular remodeling. Later, an M2-dominant environment ensures fetal tolerance and growth (88-90). Before labor, M1 macrophages reemerge to initiate an inflammatory response necessary for childbirth. Imbalanced macrophage polarization is linked to complications such as preeclampsia, preterm labor, and fetal growth restriction (93-96). Maternal obesity significantly impacts the function and phenotype of macrophages during pregnancy, contributing to immune dysregulation at both systemic and placental levels. Obesity alters monocyte activation, shifting them toward an immune-tolerant state by suppressing genes involved in interferon signaling, reactive oxygen species responses, and inflammatory activation (97). Monocytes from obese pregnant women fail to show the expected inflammatory response to LPS stimulation in late pregnancy, lack upregulation of activation markers, and do not undergo chromatin remodeling necessary for enhanced immune responses. Additionally, obesity leads to increased expression of markers associated with insulin resistance in monocytes, further complicating metabolic and immune adaptations during pregnancy (97).

At the placental level, obesity influences macrophage accumulation and polarization, leading to an increased presence of monocyte-derived macrophages while reducing the proportion of pro-inflammatory decidual macrophages (98, 99) (Figure 3). This shift is believed to be a compensatory response to mitigate obesity-induced inflammation. However, studies suggest that obesity still promotes a pro-inflammatory state, as placental macrophages exhibit heightened secretion of inflammatory cytokines, including TNF-α, IL-6, and IL-1β, particularly in response to immune challenges (71, 99-101). Given these effects, maternal obesity is strongly linked to pregnancy complications such as GD, preeclampsia, and preterm birth, emphasizing the critical role of macrophages in mediating the inflammatory and metabolic consequences of obesity during gestation. Further research is needed to understand how placental immune homeostasis impacts fetal hematopoiesis and immune system development in the offspring.

## Metabolic programming of adipose tissue macrophages by maternal obesity

The ontogeny of adipose tissue macrophages (ATMs) begins during embryonic development and continues throughout life, influenced by both intrinsic genetic programming and environmental factors such as maternal diet and nutrient availability (reviewed in (102-104)). ATMs arise from two primary sources: erythro-myeloid progenitors (EMPs) originating from the yolk sac and HSCs that later emerge in the fetal liver and bone marrow (105). EMP-derived macrophages appear first, emerging at approximately embryonic day 7 (E7) in mice and around week 3 in human gestation. These progenitors migrate to various tissues, including adipose depots, and establish the first wave of tissue-resident macrophages, often referred to as yolk-sac macrophages (39). By embryonic day 8.5 to E10, circulating EMPs infiltrate developing tissues, giving rise to macrophages that play essential roles in tissue remodeling and immune regulation (39). Around E10.5, HSCs emerge from the aorta-gonad-mesonephros region, migrate to the FL, and differentiate into fetal monocytes. By E13.5, these monocytes begin colonizing tissues, including adipose depots, contributing to the ATM pool and establishing a long-lived population of self-renewing resident macrophages (39). After birth, additional ATMs are recruited from circulating bone marrow-derived monocytes, particularly in response to metabolic demands and inflammatory stimuli. During the neonatal period, adipose tissue continues to develop, and the interplay between resident ATMs and infiltrating monocytes shapes the immune environment of the tissue (106).

Under normal physiological conditions, ATMs in lean, healthy adipose tissue primarily support lipid metabolism, tissue remodeling, and thermogenesis. However, exposure to HFD, obesity, and inflammation leads to increased recruitment of monocyte-derived ATMs, many of which adopt a proinflammatory phenotype that contributes to insulin resistance and metabolic dysfunction (107, 108) (Figure 3). Recent single-cell transcriptomics studies have identified two major ATM populations that arise from distinct developmental pathways. The first, perivascular macrophages (PVMs), are primarily derived from embryonic yolk-sac macrophages or FL monocytes and are self-renewing. These macrophages maintain vascular function, clear apoptotic cells, and regulate immune responses, contributing to adipose tissue homeostasis. The second, lipid-associated macrophages (LAMs), are mainly derived from bone marrow monocytes that infiltrate adipose tissue in response to lipid accumulation (109). LAMs express markers such as TREM2 and CD9, which are associated with lipid metabolism and inflammation. In obesity, LAMs form crown-like structures (CLS) around dying adipocytes, aiding in lipid clearance but also contributing to chronic low-grade inflammation and metabolic dysfunction (107).

The developmental trajectory of ATMs is strongly influenced by early-life environmental factors, particularly maternal diet. A high maternal intake of omega-6 fatty acids has been shown to promote an inflammatory ATM phenotype, increasing the offspring’s susceptibility to obesity and metabolic disease (110-112). Conversely, omega-3 fatty acids support an anti-inflammatory ATM profile, which enhances metabolic health by improving lipid metabolism and reducing inflammation (113, 114). ATMs exhibit remarkable plasticity, meaning they can transition between different functional states depending on environmental cues. While early in life, ATMs predominantly support metabolic and immune homeostasis, chronic metabolic stress, such as obesity, can reprogram them toward a pro-inflammatory phenotype. This shift contributes to sustained inflammation, insulin resistance, and metabolic disorders. Understanding the ontogeny of ATMs, particularly their early developmental programming, is crucial for identifying therapeutic targets that may help prevent metabolic diseases, including obesity and type 2 diabetes, later in life.

## Metabolic programming of fetal HSPCs by maternal obesity

NHP studies showed that maternal WSD induced long-term proinflammatory programming in fetal bone marrow and fetal liver CD34+ HSPCs through transcriptional and epigenetic modifications. Increased chromatin accessibility in inflammatory gene regions (FOS/JUN, NF-κB, C/EBPβ, STAT6) led to persistent immune activation, while metabolic shifts in HSPCs and their progeny favored glycolysis over oxidative phosphorylation, reinforcing an inflammatory phenotype (59) (Figure 2). Maternal WSD also skewed HSPC differentiation toward the myeloid lineage, resulting in an increased proportion of HSPCs but a reduction in erythroid progenitors (59). Additionally, maternal gut microbiome dysbiosis likely contributed to immune priming (60, 115), while elevated oleic acid in fetal and juvenile hematopoietic tissues, combined with increased bone marrow adiposity, further exacerbated inflammation (59). Collectively, these findings suggest that maternal diet-driven immune reprogramming predisposes offspring to chronic inflammatory conditions (Figure 3).

Single-cell RNA sequencing of fetal bone marrow HSPCs from maternal WSD-exposed fetuses revealed significant transcriptional changes, including upregulation of inflammatory pathways such as Toll-like receptor (TLR), TNF-α, and NOD-like receptor (NLR) signaling. Key proinflammatory genes (S100A8/9, NFKB1A, PTGS2) were highly expressed in primitive HSCs and CMPs, promoting a myeloid-biased immune response (60). Together, these findings highlight the profound impact of maternal diet on fetal HSPC development, altering metabolic programming, inflammatory potential, and regenerative function in a way that may predispose offspring to immune dysfunction and chronic disease later in life.

While the mechanisms contributing to altered developmental hematopoiesis in women with obesity remain elusive, enhanced fetal growth (macrosomia), characterized as large for gestational age (LGA), has been shown to impact HSPCs (116) (Figure 3). Research indicates that LGA neonates exhibit DNA hypermethylation in key regulatory regions of cord blood CD34+ HSPCs. These modifications affect transcription factors such as EGR1, KLF2, SOCS3, and JUNB, which are essential for maintaining stem cell quiescence and differentiation potential. Single-cell transcriptomic and chromatin accessibility analyses further reveal that these epigenetic changes correlate with decreased expression of genes involved in stem cell self-renewal, as well as reduced chromatin accessibility in critical regulatory regions (116). This study is in line with the earlier report showing intrauterine growth restriction (IUGR) and LGA births influence the epigenetic programming of cord blood CD34+ HSPCs, including a global shift towards DNA hypermethylation in both IUGR and LGA groups. This epigenetic response exhibited sexual dimorphism, with IUGR males and LGA females showing the most significant alterations. The DNA methylation changes were enriched in regulatory regions affecting genes involved in glucose homeostasis and stem cell function (117). These studies emphasize the importance of early-life epigenetic programming and its sex-specific consequences, highlighting the need for further research into how fetal growth extremes contribute to age-related diseases (62, 116, 118).

## Metabolic programming of fetal HSPCs by gestational diabetes mellitus

GD is a pregnancy complication associated with adverse health outcomes for both mothers and offspring, with long-term consequences such as increased risks of metabolic diseases and atherosclerosis in adult offspring (119-122). While maternal glucose control has mitigated some perinatal risks, the mechanisms driving these transgenerational effects remain unclear (122). Recent study in mice showed that GD can induce long-term memory in primitive HSCs of offspring (123). In this study, researchers developed two independent GD mouse models: a genetic model using Ins2Akita/+ mice, which have a mutation leading to insulin deficiency, and a pharmacological model using streptozotocin (STZ) injections to induce diabetes during pregnancy. This metabolic memory led to a skewed myeloid lineage expansion and increased susceptibility to atherosclerosis in adulthood. Mechanistically, the acquisition of this memory was linked to the activation of the advanced glycation end product receptor (AGER) and the nucleotide-binding oligomerization domain-like receptor family pyrin domain-containing 3 (NLRP3) inflammasome. These pathways promoted placental inflammation, which in turn induced epigenetic changes in HSPCs, including upregulation of DNA methyltransferase 1 (DNMT1) (123). These epigenetic modifications were associated with long-term dysregulation of the immune system and hematopoiesis, accelerated atherosclerosis development when challenged with a HFD. Despite having normal metabolic profiles in adulthood, GD offspring displayed altered immune responses, including reduced inflammatory cytokine production in response to infections. These changes suggested that GD leads to a long-term functional imprint on the hematopoietic system, affecting immune and inflammatory responses in adulthood (123). This study aligns with an earlier report showing that STZ-induced maternal hyperglycemia during the last trimester in rat dams led to an increase in myeloid progenitors in male offspring but not in female offspring exposed to a HFD (124).

Human studies have primarily focused on the impact of GD on umbilical cord blood, which represents fetal circulation and serves as a key source of HSPCs. One study reported that GD in women is associated with an increased proportion of CD34+ HSPCs in cord blood (125). Another study found that pregnant women with GD exhibit elevated numbers of CD34+ HSPCs and CMPs but a reduced number of CLPs in cord blood (126). In contrast, some studies suggest that GD is linked to a decrease in CD34+ HSPC numbers in cord blood (127, 128). The lack of a complete understanding of how GD and maternal obesity impact developmental hematopoiesis highlights significant challenges in clinical studies and underscores the importance of developing translationally relevant animal models to study these effects in settings that closely mimic human pregnancy and the pathophysiology of the response to metabolic challenges during gestation.

## Conclusions and future directions

In conclusion, the impact of maternal obesity and gestational diabetes on the differentiation of fetal HSCs underscores the intricate relationship between maternal health and offspring development. However, there remains a significant gap in understanding how maternal obesity influences distinct stages of HSPC development, including the emergence of definitive hematopoiesis in the AGM region, the migration dynamics of HSPCs from primary hematopoietic sites to the FL and bone marrow, and the specific contributions of maternal diet, metabolic status, and adiposity to developmental hematopoiesis. A deeper understanding of these mechanisms is crucial for developing targeted interventions to mitigate the adverse effects of maternal obesity on fetal hematopoiesis and improve long-term health outcomes. Future research should focus on delineating the precise developmental pathways affected by maternal metabolic conditions and exploring potential nutritional and therapeutic strategies to promote healthy fetal hematopoiesis. Additionally, the use of novel NHP models with translational relevance will be instrumental in uncovering the effects of maternal metabolism on offspring immunity, providing critical insights for human health.
